# Efficacy of a topical product combining esafoxolaner, eprinomectin and praziquantel against bedbug (*Cimex lectularius*) experimental infestations in cats[Fn FN1]

**DOI:** 10.1051/parasite/2022060

**Published:** 2022-12-21

**Authors:** Eric Tielemans, Carin Rautenbach, JF Besselaar, Frederic Beugnet

**Affiliations:** 1 Boehringer-Ingelheim Animal Health 29 avenue Tony Garnier 69007 Lyon France; 2 Clinvet International (Pty) Ltd. P.O. Box 11186, Universitas Bloemfontein 9321 Republic of South Africa; 3 Clindata International (Pty) Ltd. P.O. Box 11186, Universitas Bloemfontein 9321 Republic of South Africa

**Keywords:** Bedbug, Cat, *Cimex lectularius*, Efficacy, Esafoxolaner

## Abstract

Bedbugs (*Cimex lectularius*) are a persistent nuisance pest for humans and their home environment, and may also opportunistically feed on other mammals, including household pets like dogs and cats. NexGard^®^ Combo is a topical endectoparasiticide product for cats combining esafoxolaner, an isoxazoline compound with insecticidal and acaricidal properties, the nematocide eprinomectin and the cestocide praziquantel. The insecticidal efficacy of this product was evaluated in cats experimentally infested with *C. lectularius* in a blinded, negative controlled and randomized study. Two groups of 7 cats were formed: an untreated control group, and a NexGard^®^ Combo group treated once on Day 0 at the label dose. Cats were then challenged weekly, each with twenty unfed adult *C. lectularius*, on Days 1, 7, 14, 21 and 28. After close contact with the cat’s skin for 15 min, live fed *C. lectularius* were collected and incubated for 96 h. The weekly efficacy evaluations were based on a comparison of the number of surviving bedbugs in the control and the treated group after 48, 72 and 96 h of incubation. The model was demonstrated to be robust as in the control group the average feeding rate after the 15-minute challenge was 94%, and as 96% of incubated bed bugs were alive after 96 hours of incubation. Significant live bedbug reductions were demonstrated in the Nexgard^®^ Combo treated group: after 96 h of incubation, the reductions ranged from 80.6 to 88.0% during the Day 1 to Day 21 period, and dropped to 58% at Day 28.

## Abstract (fr)

Les punaises de lit (*Cimex lectularius*) sont une nuisance persistante pour les humains et leur environnement domestique, et peuvent également se nourrir de manière opportuniste d’autres mammifères, y compris les animaux domestiques comme les chiens et les chats. NexGard^®^ Combo est une formulation topique endectoparasiticide pour chats associant l’esafoxolaner, un composé d’isoxazoline à propriétés insecticides et acaricides, le nématodicide éprinomectine et le cestodicide praziquantel. L’efficacité insecticide de ce produit a été évaluée chez des chats infestés expérimentalement par *C. lectularius* dans une étude en aveugle et randomisée avec contrôle négatif. Deux groupes de 7 chats ont été formés, un groupe témoin non traité et un groupe traité par NexGard^®^ Combo une fois au jour 0 à la dose indiquée sur l’étiquette. Les chats ont ensuite été testés chaque semaine, chacun avec vingt *C. lectularius* adultes non nourris, les jours 1, 7, 14, 21 et 28. Après un contact étroit avec la peau du chat pendant 15 minutes, les *C. lectularius* nourris et vivants ont été collectés et incubés pendant 96 heures. Les évaluations hebdomadaires d’efficacité ont été basées sur une comparaison du nombre de punaises de lit survivantes entre le groupe témoin et le groupe traité après 48, 72 et 96 heures d’incubation. Le modèle s’est avéré robuste car dans le groupe témoin, le taux d’alimentation moyen après 15 minutes de contact était de 94 % et 96 % des punaises de lit incubées étaient vivantes après 96 heures d’incubation. Des réductions significatives du nombre de punaises de lit vivantes ont été démontrées dans le groupe traité par Nexgard^®^ Combo : après 96 heures d’incubation, les réductions variaient de 80,6 à 88,0 % pendant la période du jour 1 au jour 21 et ont chuté à 58 % au jour 28.

## Introduction

Bedbugs are obligate hematophagous insects belonging to the Hemiptera order and the Cimicidae family. The genus Cimex feeds on vertebrates including humans, birds and bats. *Cimex lectularius* and *C. hemipterus* are the two species of human medical importance, originally ectoparasites of bats, they adapted to hominids through close proximity in caves during the prehistoric era [1]. *Cimex lectularius* is predominant worldwide in all climatic regions, whereas *C. hemipterus* is mainly reported in equatorial and tropical regions [1]. A permanent pest of humans, bedbug incidence decreased after World War II through the use of DDT insecticides. However, a significant bedbug resurgence has been observed since the 1990s, mainly explained by the DDT ban in the 1970s, as well as by a rise in resistance to other insecticide products and an increase in global travel and trade [16].

Bedbugs represent a major nuisance for humans as their bites induce cutaneous manifestations such as papules, erythema, pruritus and pain, as well as psychological disorders [6, 8, 21, 26, 27, 31]. Even though bedbugs are suspected of being vectors of pathogens due to their blood feeding behavior and the fact that they carry recognised human pathogens, including bacteria, viruses, fungi, filarial nematodes and protozoans, their role as a vector remains unclear [7, 14, 22, 29, 30, 32].

Adult *C. lectularius* are 4–7 mm long, wingless, flat and oval insects. Both males and females are hematophagous. In optimal temperate conditions, eggs are produced and hatch into nymphs of 1–3 mm a few days after blood meal and mating. Five nymphal stages occur before adult emergence, each molt requiring a blood meal and taking 3 to 7 days. Adult bedbugs can live without a blood meal for many months and even for 1 to 2 years in cold environments.

Bedbugs are generally active in the dark and avoid light [7]. They hide in dark places, such as bed covers, human clothes, mattresses, bed frames, cracks, crevices, sofas, carpets, and wallpaper [21, 26]. Bedbug burden is facilitated by overcrowding and poor hygienic conditions. Bedbugs have low inherent capacity for dispersion, but disperse through their hosts’ and roosts’ movements [7].

Eliminations of bed bugs from a contaminated site can be challenging as they mostly live off their host, and are difficult to reach with cleaning and vacuum devices as well as insecticide products. Eradication may require intensive measures, including non-chemical actions such as heating or freezing contaminated items, dismantling furniture and parts of the home environment, and chemical actions through intensive and long-lasting use of insecticide products [8, 19, 26]. Furthermore insecticide resistance in bed bugs is an increasing reality [3, 5, 9, 25].

In veterinary medicine, beside the strategies of environmental control through hygienic, physical and chemical measures, the administration of insecticide medications to an animal host is a widely and successfully used strategy, for example for the control of flea infestations in dogs and cats [15]. In human medicine, other than the use of repellent contact substances against flying insects, the on-host use of insecticide products is not a common strategy.

Data about efficacy of systemic insecticides affecting bedbugs through their blood meal are limited. *In vitro* demonstrations of activity on bedbug survival were demonstrated for moxidectin, a systemic macrocyclic lactone [38], and for spinosad, a tetracyclic macrolide, and fluralaner, an isoxazoline [33]. More recently the efficacy of afoxolaner, an isoxazoline, was demonstrated against *C. lectularius* feeding on treated dogs, in an experimental model [2].

Isoxazoline is a family of insecticide and acaricide systemic molecules developed for domestic animals and includes afoxolaner, esafoxolaner, fluralaner, sarolaner and lotilaner. Afoxolaner and esafoxolaner are characterized by high plasma protein binding and a long half-life, ensuring high level and long-lasting efficacy against hematophagous arthropods [18, 34].

NexGard^®^ Combo, a topical combination of esafoxolaner, eprinomectin and praziquantel was developed for cats with the aim to offer a wide spectrum of antiparasitic activity, including insects, Acari, nematodes and cestodes [12, 20, 36, 37]. Esafoxolaner is the purified and active (S)-enantiomer of afoxolaner, the racemic form. Afoxolaner is available in two oral products for dogs with indications against flea, tick and mite infestations [10, 11]. Afoxolaner was also demonstrated to be efficacious against flying hematophagous insects that do not live on the host, i.e. the sandfly (*Phlebotomus perniciosus*) [28], mosquito (*Aedes aegypti*) [23], stable fly (*Stomoxys calcitrans*) [35], kissing bug (*Triatoma infestans*) [24] and bedbug (*C. lectularius*) [2]. Eprinomectin, the nematocide compound, and praziquantel, the cestocide compound of the product, have insignificant and no potency on arthropods, respectively, when administered in combination with esafoxolaner (unpublished internal data).

This manuscript describes a study conducted to verify whether *C. lectularius,* an ectoparasite of bats and humans, may also feed on cats in an artificial infestation context, and to verify the insecticidal potency of NexGard^®^ Combo against *C. lectularius* fed on treated cats.

## Materials and methods

### Ethics

The study plan was reviewed and approved by the Sponsor’s and local institutional animal care and use committee. The study was conducted in accordance with the European Union Directive 2010/63/EU on the protection of animals used for scientific purposes [13].

### Study design

The study was conducted in accordance with Good Clinical Practices [17].

The study was conducted under a negative-controlled, masked and randomized design. A pre-treatment bedbug challenge was conducted with 16 cats during the 7-day acclimatization period for selection of adequate cats and randomization to study groups. Two blocks were formed on the basis of live fed bedbug counts after a 15-minute challenge and 48 h of incubation (the two cats with lowest live fed bedbug count at 48 h were removed). The fourteen selected cats were randomized to a negative control sham-treated group and to a NexGard^®^ Combo treated group of 7 cats each. None of the cats had been treated with an ectoparasiticide product over the 12 weeks preceding Day 0. Animal and randomization details are provided in Table 1.

**Table 1:** Animal, and randomization details.

Group	Sex	Age (months) on Day -3	Body weight (kg) on Day -3	Number and status of bed bugs 48 h after Day -6 challenge		
				Live fed	Live unfed	Dead
Sham-dose	Female	37	3.2	18	2	0
	Female	45	3.2	17	3	0
	Male	13	2.9	18	2	0
	Male	27	4.1	20	0	0
	Male	43	5.0	18	2	0
	Male	14	3.9	19	1	0
	Male	13	3.2	20	0	0
NexGard Combo	Female	53	3.2	17	3	0
	Female	42	4.4	18	2	0
	Female	19	2.8	19	1	0
	Female	14	3.2	19	1	0
	Female	13	2.6	19	1	0
	Male	30	5.1	17	3	0
	Male	13	3.2	20	0	0

All cats were healthy, DSH, purpose-bred.

On Day 0, cats assigned to the NexGard^®^ Combo group were topically treated with an applicator of 0.9 mL, providing 10.8 mg esafoxolaner, 3.6 mg eprinomectin and 74.7 mg praziquantel, following label instructions. The administered dosage of esafoxolaner ranged from 2.1 to 3.9 mg/kg.

Cats were observed daily from Day -7 to Day 28, and hourly for 4 h after sham-dose or NexGard^®^ Combo administration, for general health and adverse reactions. Personnel involved with evaluation of safety and efficacy were unaware of treatment group assignments.

Cats were challenged with *C. lectularius* on Day -6 for selection and randomization, and on Days 1, 7, 14, 21 and 28 for insecticidal activity evaluations.

### *Cimex lectularius* experimental infestation model

The *C. lectularius* had been collected from human habitats in Bloemfontein (Orange Free State, South Africa) in 2017 (this study was conducted in 2021). Since collection, they had been maintained in an insectarium and fed weekly on rabbits using described methods [4]. Adult *C. lectularius*, kept unfed since their last molt from N_5_ to adult, were used in the challenges (they had been unfed for at least 5 days and at most 14 days, as the N_5_ molted to adults in 5 to 7 days after their blood meal, and the adults were kept for a maximum of 7 days).

On Day -7, the first day of acclimatization, each cat had an area of their haircoat, measuring approximately 10 cm in diameter, on the flanks, caudal to the ribs at approximately 15 cm from the application site, shaved on the right and the left side in preparation for the challenges. Cats were challenged with 20 *C. lectularius* on Day -6 for selection and randomization, and on Days 1, 7, 14, 21 and 28 for insecticidal activity evaluations. For each challenge, cats were sedated with a 0.12 mL/kg intramuscular injection of Domitor^®^ (medetomidin 1 mg/mL). The sedated cats were placed in a dark room to facilitate the feeding of the bedbugs. Two chambers, covered with an appropriate mesh netted material through which the bedbugs could feed, containing ten *C. lectularius* adults each (5 males and 5 females), were each held against the shaved areas of the cat on the left and right side for 15 min, by applying light pressure to ensure sufficient contact with the cat’s skin. After each feeding phase, the cat’s skin was examined for any abnormality and sedation reversed with a 0.06 mL/kg intramuscular injection of Antisedan^®^ (atipamezole 5 mg/mL) when deemed necessary. Feeding assessment of bedbugs was performed immediately post-challenge by visual observation of their abdomen. Bedbugs were only classified as fed when it was clear that a blood meal had been taken. The classification was easier with males, as their abdomen appeared to elongate with the blood meal and as they fed quickly in comparison to females (author’s observation). With females, the engorgement was not always as clear as it did not change the shape of the abdomen and as they seemed to feed slower during the 15-minute contact period (author’s observation). The collected live fed bedbugs were then incubated in an insectarium (temperature: 22.1 to 36.2 °C, humidity: 62.1 to 82.1%) and viability assessments were performed after 48, 72 and 96 h.

### Efficacy evaluations

The efficacy against bedbugs was calculated for each incubation duration (48 h, 72 h and 96 h) in relation to each challenge day (Days 1, 7, 14, 21 and 28) according to the formula given below. Efficacy calculations were based on arithmetic mean values.

Efficacy (%) = 100 × (Mc – Mt)/Mc,

where:

Mc = Proportion* of live, fed bedbugs in the control group at 48, 72 and 96 h, after the 15 min challenge phase;
Mt = Proportion* of live, fed bedbugs in the treated group at 48, 72 and 96 h, after the 15 min challenge phase.
*mean proportion of collected live bedbugs after the 15-minute challenge phase in relation to the 20 live unfed bedbugs used for the challenge.

The groups were compared using an ANOVA (Proc GLM procedure in SAS) with a treatment effect on logarithmic transformed fed bedbug (count + 1) data. SAS Version 9.4 was used for all the statistical analyses. The level of significance of the formal tests was set at 5%, all tests were two sided.

## Results

No adverse reactions to the feeding and bites of the bedbugs or to the treatment were observed. No abnormal signs were observed during the daily health observations or during the post-treatment observations.

The incubation data and efficacy results are described in Table 2.

**Table 2:** Efficacy.

		Challenge Day 1				Challenge Day 7				Challenge Day 14				Challenge Day 21				Challenge Day 28			
Group		15 min	48 h	72 h	96 h	15 min	48 h	72 h	96 h	15 min	48 h	72 h	96 h	15 min	48 h	72 h	96 h	15 min	48 h	72 h	96 h
Sham-dose	AM	18.9	18.7	18.7	18.1	19.1	17.9	17.9	16.4	18.6	18.6	18.3	18.1	19.0	19.0	19.0	19.0	18.4	18.4	18.4	18.3
	Prop.		0.99	0.99	0.96		0.93	0.93	0.86		1.00	0.98	0.98		1.00	1.00	1.00		1.00	1.00	0.99
NexGard Combo	AM	16.7	11.0	3.7	3.1	18.9	12.0	3.6	3.1	15.4	5.9	3.9	2.6	15.7	7.4	3.7	1.9	17.00	13.0	7.7	7.0
	Prop.		0.66	0.22	0.19		0.64	0.19	0.17		0.39	0.26	0.18		0.48	0.24	0.12		0.77	0.45	0.41
	**Efficacy (%)**		**33.3**	**78.1**	**80.6**		**31.3**	**79.8**	**80.6**		**61.0**	**74.0**	**81.8**		**51.8**	**76.1**	**88.0**		**23.3**	**54.5**	**58.2**
	*P*-value		0.0025	<.0001	<.0001		0.0064	<.0001	<.0001		<.0001	<.0001	<.0001		<.0001	<.0001	<.0001		0.0004	<.0001	<.0001

AM = arithmetic mean of live fed bedbugs, collected and placed in incubation (15 min), and surviving (48 h, 72 h and 96 h).

Prop. = proportion of live bedbugs relative to the number of bedbugs placed in incubation.

Efficacy (%) = 100 × (Mc – Mt)/Mc, where Mc = Proportion of live, fed bedbugs in the control group at 48 h, 72 h and 96 h, Mt = Proportion of live, fed bedbugs in the treated group at 48 h, 72 h and 96 h, after the 15 min challenge phase.

*P*-value = One-way ANOVA with a treatment effect.

The model was demonstrated to be robust, as in the control group, the average feeding rate after the 15-minute challenges was 94% (i.e. inclusive of the 5 challenges, out of 20 bedbugs a mean 18.8 bedbugs had fed after 15 min, and none had died), and as 98%, 98% and 96% of the incubated bedbugs were alive after 48, 72 and 96 h, respectively.

Significant live bedbug reductions ranging over the month from 23 to 61% after 48 h of incubation, from 55 to 80% after 72 h of incubation, and from 58 to 88% after 96 h of incubation were observed in the Nexgard^®^ Combo treated group. The durations of incubation were important, as even though all reduction results were statistically significant after 48 h of incubation, the percent reductions became biologically significant after 72 h of incubation, as they consistently exceeded 55%.

## Discussion

It is unknown whether in real life *C. lectularius* bedbugs readily feed on cats, but comparable results of host feeding were demonstrated in dogs in a similar study design, where the average feeding rate after 15-minute challenges was ~95% [2]. The fact that bedbugs are being maintained in laboratory per weekly feeding on rabbits [4], and the high feeding rates in cats demonstrated in this study, support the hypothesis that bedbugs may have adapted to mammalian hosts beyond humans and bats and may opportunistically feed on pets. The hypothesis of host compatibility to species other than humans and bats is also sustained by the high survival rates of *C. lectularius* fed with cat, dog and rabbit blood.

In view of the significant 4-week reduction of bedbugs demonstrated in this study after experimental infestations of cats treated with esafoxolaner combined with eprinomectin and praziquantel, and the comparable results demonstrated with dogs treated with afoxolaner [2], it may be hypothesised that pets treated with these systemic insecticide compounds may bring a complementary contribution to the control of *C. lectularius* environmental contaminations. This hypothesis is further supported by the fact that, unlike flying insects, bedbugs do not disperse from their roost and thus have a host interaction restricted to mammals present in the same home environment, increasing the probability of feeding several times on the same host during their life. Nevertheless, to support this hypothesis, it will be necessary to demonstrate that bedbugs feed on pets in natural environments, and feed on pets despite the presence of humans, the preferred host. If these assumptions are verified, it will also be necessary to confirm that when bedbugs feed on NexGard^®^ Combo treated cat in natural challenges, the efficacy remains, as the contact site for bedbug feeding in this study was limited and may have been influenced by its proximity to the application site (even though esafoxolaner is transdermally absorbed and acts mostly through a systemic pathway). Ultimately, it will be necessary to demonstrate a significant effect of treated pets in a contaminated household environment. Other hypotheses such as the efficacy of these isoxazoline compounds after a second blood meal, or their efficacy on blood feeding larval stages, or the survival of fed bedbugs after longer incubation periods, may also be interesting to verify, to better understand the benefits of this approach.

Bedbug environmental contaminations represent a significant concern for public health worldwide. The strategies for environmental bedbug control and eradication are based on physical elimination of the insects by hygiene measures, and the use of environmental chemical pesticides. Successful bedbug elimination from a contaminated site can be highly challenging as they mostly live off their host, and are difficult to reach with cleaning devices and insecticide products. An approach based on treating potential hosts with preventive insecticides would provide an important complement to break the life cycle of this insect and significantly improve the control measures. In the absence of preventive insecticide use in humans, the main host, the one-health approach of systemic insecticide treatment of pets, an opportunist host, may bring an innovative complement to the eradication strategies of household bedbug contaminations.

## Competing interest

The work reported herein was funded by Boehringer Ingelheim Animal Health. Some of the authors are current employees of Boehringer Ingelheim Animal Health or at external organizations. Other than that, the authors declare no conflict of interest. This document is provided for scientific purposes only. Any reference to a brand or trademark herein is for information purposes only and is not intended for any commercial purposes or to dilute the rights of the respective owners of the brand(s) or trademark(s). NexGard Combo^®^ is a registered trademark of the Boehringer Ingelheim Group.

## References

[1] Akhoundi M, Sereno D, Durand R, Mirzaei A, Bruel C, Delaunay P, Marty P, Izri A. 2020. Bed bugs (Hemiptera, Cimicidae): Overview of classification, evolution and dispersion. International Journal of Environmental Research and Public Health, 2020(17), 4576. 10.3390/ijerph17124576 PMC7345932 32630433

[2] Beugnet F, Rautenbach C, van der Mescht L, Lebon W, Aouiche N, Liebenberg J. 2021. Insecticidal efficacy of afoxolaner against bedbugs, *Cimex lectularius,* when administered orally to dogs. Parasite, 28, 7. 33528356 10.1051/parasite/2021004 PMC7852378

[3] Candy K, Akhoundi M, Bruel C, Izri A. 2018. Ineffectiveness of insecticide bendiocarb against a *Cimex lectularius* (Hemiptera: Cimicidae) population in Paris, France. Journal of Medical Entomology, 55(6), 1648–1650. 32478830 10.1093/jme/tjy126

[4] Cannet A, Akhoundi M, Berenger JM, Michel G, Marty P, Delaunay P. 2015. A review of data on laboratory colonies of bed bugs (Cimicidae), an insect of emerging medical relevance. Parasite, 2015(22), 21. 10.1051/parasite/2015021 PMC4475256 26091944

[5] Dang K, Doggett SL, Singham GV, Lee CY. 2017. Insecticide resistance and resistance mechanisms in bed bugs, *Cimex* spp. (Hemiptera: Cimicidae). Parasites & Vectors, 10, 318. 28662724 10.1186/s13071-017-2232-3 PMC5492349

[6] Davies TG, Field LM, Williamson MS. 2012. The re-emergence of the bed bug as a nuisance pest: implications of resistance to the pyrethroid insecticides. Medical and Veterinary Entomology, 26(3), 241–254. 22235873 10.1111/j.1365-2915.2011.01006.x

[7] Delaunay P, Blanc V, Del Giudice P, Levy-Bencheton A, Chosidow O, Marty P, Brouqui P. 2011. Bedbugs and Infectious Diseases. 2011. Clinical Infectious Diseases, 52, 200–210. 21288844 10.1093/cid/ciq102 PMC3060893

[8] Doggett SL, Dwyer DE, Peñas PF, Russell RC. 2012. Bed bugs: clinical relevance and control options. Clinical Microbiology Reviews, 25(1), 164–192. 22232375 10.1128/CMR.05015-11 PMC3255965

[9] Durand R, Cannet A, Berdjane Z, Bruel C, Haouchine D, Delaunay P, Izri A. 2012. Infestation by pyrethroids resistant bed bugs in the suburb of Paris, France. Parasite, 2012(19), 381–387. 10.1051/parasite/2012194381 PMC3671460 23193523

[10] EMA. 2013. CVMP assessment report for NexGard (EMEA/V/C/002729/0000). https://www.ema.europa.eu/en/documents/assessment-report/nexgard-epar-public-assessment-report_en.pdf.

[11] EMA. 2014. CVMP Assessment Report for NEXGARD SPECTRA (EMEA/V/C/003842/0000). https://www.ema.europa.eu/en/documents/assessment-report/nexgard-spectra-epar-public-assessment-report_en.pdf.

[12] EMA. 2020. CVMP Assessment Report for NEXGARD COMBO (EMEA/V/C/005094/0000). https://www.ema.europa.eu/en/documents/assessment-report/nexgard-combo-epar-public-assessment-report_en.pdf.

[13] European Union. 2010. Directive 2010/63/EU of the European Parliament and of the Council of 22 September 2010 on the protection of animals used for scientific purposes. Official Journal of the European Union, 33–79.

[14] Goddard J, deShazo R. 2009. Bed bugs (*Cimex lectularius*) and clinical consequences of their bites. JAMA, 301(13), 1358–1366. 19336711 10.1001/jama.2009.405

[15] Halos L, Beugnet F, Cardoso L, Farkas R, Franc M, Guillot J, Pfister K, Wall R. 2014. Flea control failure? Myths and realities Trends in Parasitology, 30(5), 228–233. 24661796 10.1016/j.pt.2014.02.007

[16] Hwang SW, Svoboda TJ, De Jong IJ, Kabasele KJ, Gogosis E. 2005. Bed Bug Infestations in an Urban Environment. Emerging Infectious Diseases, 2005, 11. 10.3201/eid1104.041126 PMC3320350 15829190

[17] International Cooperation on Harmonization of Technical Requirements for Registration of Veterinary Medicinal Products (VICH). 2000. VICH Guideline 9, Good Clinical Practice. https://www.vichsec.org/en/guidelines/pharmaceuticals/pharma-efficacy/good-clinical-practice.html . 10.20506/rst.31.1.2113 22849284

[18] Jacquot V, Buellet P, Letendre L, Tong W, Li H, Tielemans E. 2021. Pharmacokinetics of a novel endectoparasiticide topical formulation for cats, combining esafoxolaner, eprinomectin and praziquantel. Parasite, 28, 19. 33812451 10.1051/parasite/2021014 PMC8019567

[19] Jourdain F, Delaunay P, Bérenger JM, Perrin Y, Robert V. 2016. The common bed bug (*Cimex lectularius*) in metropolitan France. Survey on the attitudes and practices of private- and public-sector professionals. Parasite, 23, 38. 27605306 10.1051/parasite/2016038 PMC5018931

[20] Knaus M, Baker C, Alva R, Mitchell E, Irwin J, Shukullari E, Veliu A, Ibarra-Velarde F, Liebenberg J, Reinemeyer C, Tielemans E, Wakeland K, Johnson C. 2021. Efficacy of a novel topical combination of esafoxolaner, eprinomectin and praziquantel in cats against *Toxocara cati* and *Dipylidium caninum* . Parasite, 28, 28. 33812460 10.1051/parasite/2021024 PMC8019557

[21] Kolb A, Needham GR, Neyman KM, High WA. 2009. Bedbugs. Dermatology and Therapy, 22, 347–352 10.1111/j.1529-8019.2009.01246.x 19580578

[22] Lai O, Ho D, Glick S, Jagdeo J. 2016. Bedbugs and possible transmission of human pathogens: a systematic review. Archives of Dermatological Research, 308(8), 531–538. 27295087 10.1007/s00403-016-1661-8 PMC5007277

[23] Liebenberg J, Fourie J, Lebon W, Larsen D, Halos L, Beugnet F. 2017. Assessment of the insecticidal activity of afoxolaner against *Aedes aegypti* in dogs treated with NexGard®. Parasite, 24, 39. 29063828 10.1051/parasite/2017042 PMC5654328

[24] Loza A, Talaga A, Herbas G, Jair Canaviri R, Cahuasiri T, Luck L, Guibarra A, Goncalves R, Pereira JA, Gomez SA, Picado A, Messenger LA, Bern C, Courtenay O. 2017. Systemic insecticide treatment of the canine reservoir of *Trypanosoma cruzi* induces high levels of lethality in *Triatoma infestans*, a principal vector of Chagas disease. Parasites & Vectors, 2017(10), 344. 10.1186/s13071-017-2278-2 PMC5518140 28724448

[25] Moshaverinia A, Raouf-Rahmati A, Jarahi L, Bergquist R, Zorrilla-Vaca A, Kiani F, Jadidoleslami A, Doggett SL, Zarean M, Majma A, Reza Youssefi M, Moghaddas E, Kiani B. 2022. Geographical patterns and mechanisms of *Cimex lectularius Linnaeus*, 1758, and *Cimex hemipterus Fabricius*, 1803 (Hemiptera: Cimicidae) resistance to insecticides: a systematic review and meta-analysis. Parasitology Research, 121(7), 1817–1827. 35524788 10.1007/s00436-022-07530-7

[26] Parola P, Izri A. 2020. Bedbugs. The New England Journal of Medicine, 382(23), 2230–2237. 32492304 10.1056/NEJMcp1905840

[27] Peres G, Buonalumi Tacito Yugar L, Haddad Junior V. 2018. Breakfast, lunch, and dinner sign: a hallmark of flea and bedbug bites. Annals Brasilian Dermatology, 2018(93), 759–760. 10.1590/abd1806-4841.20187384 PMC6106677 30156636

[28] Perier N, Lebon W, Meyer L, Lekouch N, Aouiche N, Beugnet F. 2019. Assessment of the insecticidal activity of oral afoxolaner against *Phlebotomus perniciosus* in dogs. Parasite, 2019(26), 63. 10.1051/parasite/2019063 PMC6830354 31687926

[29] Peta V, Pietri JE. 2021. Experimental infection of bed bugs (*Cimex lectularius* L.) with *Burkholderia multivorans*. Medical and Veterinary Entomology, 35(3), 507–512. 33884648 10.1111/mve.12520

[30] Reinhardt K, Siva-Jothy MT. 2007. Biology of the bed bugs (Cimicidae). Annual Review of Entomology, 52, 351–374. 10.1146/annurev.ento.52.040306.133913 16968204

[31] Sabou M, Imperiale DG, Andrès E, Abou-Bacar A, Foeglé J, Lavigne T, Kaltenbach G, Candolfi E. 2013. Bed bugs reproductive life cycle in the clothes of a patient suffering from Alzheimer’s disease results in iron deficiency anemia. Parasite, 20, 16. 23673315 10.1051/parasite/2013018 PMC3718524

[32] Salazar R, Castillo-Neyra R, Tustin AW, Borrini-Mayorí K, Náquira C, Levy MZ. 2015. Bedbugs (*Cimex lectularius*) as vectors of *Trypanosoma cruzi*. American Journal of Tropical Medicine and Hygiene, 92(2), 331–335. 25404068 10.4269/ajtmh.14-0483 PMC4347337

[33] Sheele JM. 2020. A Preliminary report showing spinosad and fluralaner are able to incapacitate *Cimex lectularius* L., the common bed bug. Cureus, 12(4), e7529. 32377477 10.7759/cureus.7529 PMC7198093

[34] Shoop WL, Hartline EJ, Gould BR, Waddell ME, McDowell RG, Kinney JB, Lahm GP, Long JK, Xu M, Wagerle T, Jones GS, Dietrich RF, Cordova D, Schroeder ME, Rhoades DF, Benner EA, Confalone PN. 2014. Discovery and mode of action of afoxolaner, a new isoxazoline parasiticide for dogs. Veterinary Parasitology, 201, 179–189. 24631502 10.1016/j.vetpar.2014.02.020

[35] Tielemans E, Aouiche N, Saunders A, Besselaar JF, Beugnet F. 2021 Jul. Insecticidal efficacy of afoxolaner against *Stomoxys calcitrans* (Diptera: Muscidae) in dogs. Current Research in Parasitology and Vector-Borne Diseases, 21(1), 100043. 10.1016/j.crpvbd.2021.100043 PMC8906105 35284852

[36] Tielemans E, Otsuki T, Cheesman T, Selmes F, Pfefferkorn A, Prullage J. 2021. Efficacy of a novel topical combination of esafoxolaner, eprinomectin and praziquantel against fleas in cats, under field conditions. Parasite, 28, 22. 33812454 10.1051/parasite/2021018 PMC8019569

[37] Tielemans E, Prullage J, Tomoko O, Liebenberg J, Capári B, Sotiraki S, Kostopoulou D, Ligda P, Ulrich M, Knaus M. 2021. Efficacy of a novel topical combination of esafoxolaner, eprinomectin and praziquantel against ear mite (*Otodectes cynotis*) infestations in cats. Parasite, 28, 26. 33812458 10.1051/parasite/2021022 PMC8019571

[38] Zha C, Wang C, Sheele JM. 2017. Effect of moxidectin on bed bug feeding, development, fecundity, and survivorship. Insects, 2017(8), 106. 10.3390/insects8040106 PMC5746789 28973981

